# Development and validation of an interpretable machine learning model for predicting low muscle mass in patients with rheumatoid arthritis: a multicenter study

**DOI:** 10.3389/fmed.2025.1694320

**Published:** 2025-11-19

**Authors:** Feiyue Zhou, Bin Zhou, Yuan Qu, Shuai Zhong, Ting Liu, Yuan Liu, Xiaohu Zhao, Xuanhe Tian, Xiaojing Hao, Ping Jiang

**Affiliations:** 1 First College of Clinical Medicine, Shandong University of Traditional Chinese Medicine, Jinan, China; 2 Department of Orthopaedics, The Affiliated Hospital of Shandong University of Traditional Chinese Medicine, Jinan, China

**Keywords:** rheumatoid arthritis, National Health and Nutrition Examination Survey, machine learning model, low muscle mass, sarcopenia

## Abstract

**Background:**

This study aims to develop a predictive model for identifying rheumatoid arthritis (RA) patients at risk of low muscle mass using easily obtainable clinical indicators. The goal is to facilitate targeted screening for individuals at high risk of sarcopenia, optimize diagnostic strategies, reduce the burden of additional testing, and improve the efficiency of early identification and intervention.

**Methods:**

This study analyzed data from 1,260 RA patients obtained from the National Health and Nutrition Examination Survey (NHANES) database and the Affiliated Hospital of Shandong University of Traditional Chinese Medicine (SHUTCM). Eight machine learning models were developed, including Random Forest, LightGBM, XGBoost, CatBoost, Support Vector Machine (SVM), K-Nearest Neighbors (KNN), Logistic Regression, and a weighted ensemble model. Model performance was evaluated using metrics such as accuracy, area under the receiver operating characteristic curve (AUC), F1 score, Precision, Recall, and Brier score loss. The SHapley Additive exPlanation (SHAP) method was used to rank feature importance and interpret the final model.

**Results:**

Among all machine learning models, the tree-based weighted ensemble model demonstrated the best performance, achieving an AUC of 0.921, outperforming all individual models. The model exhibited good calibration and higher net clinical benefit in decision curve analysis, especially within the probability threshold range of 0.2 to 0.8, and achieved an AUC of 0.848 on the test set, demonstrating a certain degree of generalizability. SHAP analysis identified BMI, albumin, hemoglobin, age, and creatinine as the most important features for predicting the risk of low muscle mass. SHAP dependency and waterfall plots further showed the model’s decision-making mechanisms. Finally, we developed an online risk prediction calculator based on the FastAPI framework, which automatically generates individualized low muscle mass risk scores based on user input. The tool has been deployed on the Hugging Face platform and is accessible online.

**Conclusion:**

Based on a large, multicenter dataset, we developed and validated an explainable ML model capable of identifying individuals with a high risk of low muscle mass among patients with rheumatoid arthritis. This model may serve as a decision-support tool for clinicians in guiding further screening and diagnosis of sarcopenia.

## 1 Introduction

Rheumatoid arthritis (RA) is a chronic autoimmune condition marked by persistent synovitis, which may result in progressive deterioration, deformity, and disability of joints. Its global incidence rate is 0.5 to 1% (1). Although RA is primarily characterized by synovitis, long-term systemic inflammation, metabolic dysregulation, and nutritional disturbances, can lead to adverse effects beyond joint involvement (2–4). Extra-articular manifestations and complications in RA patients further increase disease burden and negatively affect both quality of life and prognosis.

Sarcopenia is a complication in RA patients. Defined as a progressive and generalized syndrome characterized by the loss of muscle strength and muscle mass, sarcopenia has a notably high prevalence among individuals with RA. Current studies have indicated that the incidence of sarcopenia in RA patients ranges from 24 to 61.7% (5–8). Sarcopenia significantly impairs physical functioning and quality of life (9, 10), and increases the risk of falls and fractures (5, 11, 12), further contributing to the disease burden in patients.

Despite growing recognition of sarcopenia among RA patients, it remains overlooked in clinical practice (13). Currently, the diagnosis of sarcopenia typically includes the assessment of muscle mass, which is primarily measured using dual-energy X-ray absorptiometry (DXA) or bioelectrical impedance analysis (BIA). However, these methods are highly dependent on specialized equipment, which not only increases the examination burden for patients but also limits their accessibility in primary healthcare settings, where such devices may not be available. These factors objectively reduce patients’ willingness to undergo screening and diagnosis for sarcopenia, thereby posing a barrier to its clinical awareness and broader implementation.

Therefore, this study aimed to develop a predictive model based on routinely available clinical data to estimate the probability of low muscle mass in RA patients. The goal is to enable targeted identification of individuals at high risk of sarcopenia, guide further screening and diagnostic efforts, and reduce the examination burden on patients.

## 2 Materials and methods

### 2.1 Study population

In this study, we included data from the National Health and Nutrition Examination Survey (NHANES) and the Affiliated Hospital of Shandong University of Traditional Chinese Medicine (SHUTCM).

NHANES data from 2001 to 2018 were analyzed in this study. NHANES is a thorough population health survey conducted by the Centers for Disease Control and Prevention (CDC) to gather health and nutrition data from the American population. It collects health and nutritional information from a representative sample of the U.S. population and includes detailed laboratory test results, health questionnaires, and mortality records. The survey received approval from the Research Ethics Review Committee of the National Center for Health Statistics (NCHS), and informed consent was collected from all participants. As these data are de-identified and publicly released by NCHS, no additional authorization or special access was required.

At the SHUTCM, we enrolled patients diagnosed with RA between August 2022 and January 2025. The study was conducted by the principles of the Declaration of Helsinki and was approved by the institutional ethics committee [Approval No. (2022)083-KY]. Informed consent was waived due to the retrospective nature of the study.

We excluded individuals under the age of 18, individuals without a confirmed RA diagnosis, those lacking essential hematological laboratory data, and those missing key skeletal muscle measurements.

### 2.2 Clinical feature assessment

The variables included in this study were gender, age, body mass index (BMI), neutrophil count, lymphocyte count, hemoglobin, platelet count, alanine aminotransferase (ALT), aspartate aminotransferase (AST), cholesterol, albumin, urea, creatinine, and uric acid.

The NHANES research gathered RA-related data via a self-administered questionnaire. Participants were asked in question MCQ160a: “Have doctors or other health professionals informed you that you have arthritis?” with possible responses being “Yes,” “No,” “Refused,” or “Do not know.” If the answer was “Yes,” they were further asked in question MCQ195: “What type of arthritis are you suffering from?” with response options including “Rheumatoid arthritis,” “Osteoarthritis,” “Psoriatic arthritis,” “Other,” “Refused,” and “Do not know.” Participants who answered “Yes” to MCQ160a and selected “Rheumatoid arthritis” in MCQ195 were identified as having RA (questionnaire available at: https://wwwn.cdc.gov/nchs/nhanes/Default.aspx).

For patients from the SHUTCM, clinical information including RA diagnosis and relevant laboratory data was extracted from electronic medical records.

To reduce variability arising from differences in laboratory instruments and reagent batches across datasets, standardization procedures were applied. The systemic immune-inflammation index (SII; where SII = platelet count × neutrophil count/lymphocyte count) and the neutrophil-to-lymphocyte ratio (NLR) were derived through computation.

### 2.3 Assessment of low muscle mass

In the NHANES population, low muscle mass was defined based on the skeletal muscle mass index (SMI), which was derived from appendicular skeletal muscle mass (ASM) obtained through DXA scans of the limbs. SMI was calculated as ASM divided by BMI. According to the Foundation for the National Institutes of Health Sarcopenia Project criteria, individuals were classified as having Low muscle mass if their SMI was less than 0.789 for men or less than 0.512 for women (14).

For patients enrolled from the SHUTCM, low muscle mass was assessed according to the criteria established by the Asian Working Group for Sarcopenia using BIA (15). ASM was obtained via BIA and adjusted for height. Low muscle mass was defined as <7.0 kg/m^2^ for men and <5.7 kg/m^2^ for women.

### 2.4 Feature engineering and data preprocessing

In the baseline characteristics table, continuous variables were expressed as mean ± standard error and compared using Student’s *t*-test. For continuous variables that did not follow a normal distribution, data were presented as median and interquartile range and assessed using the Mann–Whitney *U* test. Categorical variables were summarized as counts and percentages, and compared using the chi-square test or Fisher’s exact test, as appropriate.

We divided the RA cohort by time: participants enrolled before January 2024 were assigned to the training set, where 10-fold cross-validation was used for training and validation. Participants enrolled between January 2024 and January 2025 were assigned to the test set.

To assess heterogeneity between the two centers included in the training set, we compared the distributions of all covariates across centers. The Kolmogorov–Smirnov (K–S) test was applied to evaluate whether the distributions of continuous variables differed significantly between centers. Variables with K–S test *p*-values <0.05 were considered to have statistically significant distributional differences. Univariate and multivariate logistic regression analyses were performed to explore the associations between variables and low muscle mass. In addition, restricted cubic spline (RCS) models were used to further investigate potential nonlinear relationships between continuous variables and the risk of low muscle mass.

Because NHANES lacks RA-specific activity indices in some cycles, we additionally quantified how well hematology-derived inflammation proxies relate to acute-phase reactants in the clinical cohort. Specifically, using the SHUTCM dataset we computed Spearman’s rank correlation (*ρ*) between NLR/SII and CRP/ESR (two-sided *p*-values; 95% CIs via nonparametric bootstrap).

Data cleaning were performed prior to modeling. The target variable was the presence of low muscle mass. During the feature engineering stage, an automated interaction construction approach was applied. Several clinically relevant feature pairs (e.g., Age × BMI, Hemoglobin × Creatinine, Albumin × ALT) were used to generate new numerical interaction terms to enhance the representational capacity of the dataset. Categorical variables were encoded using one-hot encoding. To avoid data leakage, all standardization procedures for numerical variables were embedded within the cross-validation pipeline.

### 2.5 Model construction and ensemble strategy

Seven machine learning models, including Random Forest, LightGBM, XGBoost, CatBoost, Support Vector Machine (SVM), K-Nearest Neighbors (KNN), and Logistic Regression were used to predict the risk of low muscle mass in RA patients. To optimize the performance of each base model, automated hyperparameter tuning was performed using Optuna with Bayesian optimization, targeting the F1 score as the objective metric. The maximum number of optimization iterations was set to 15. Given the superior performance of tree-based models, we calculated their out-of-fold AUC scores and used them as weights to construct a weighted ensemble model, which served as the final prediction model.

### 2.6 Training strategy and cross-validation

To ensure the robustness of the evaluation results, we applied stratified 10-fold cross-validation. Within each fold, the training set was oversampled using the Synthetic Minority Over-sampling Technique (SMOTE) to address class imbalance. Each base model was independently trained on the resampled training set and generated probability predictions for the validation set. To determine the optimal classification threshold, we evaluated 999 candidate thresholds ranging from 0.001 to 0.999 for each model, selecting the one that maximized the F1 score. Given the strong performance of tree-based models, we selected four high-performing models—Random Forest, LightGBM, XGBoost, and CatBoost—and used their validation-set predicted probabilities as input features for the ensemble model. Finally, a weighted ensemble model was constructed using out-of-fold AUC-based weights from these four models to generate the final prediction. To evaluate the generalizability of the ensemble model, we conducted validation on the test set.

### 2.7 Performance evaluation and visualization

Several commonly used evaluation metrics were employed to assess the reliability of the models, including accuracy, area under the receiver operating characteristic curve (AUC), F1 score, precision, recall, and Brier score loss. To assess the clinical utility of the models under different decision-making scenarios, we plotted the receiver operating characteristic (ROC) curves, calibration curves, and decision curve analysis (DCA) plots for all models. The DCA plots included “Treat-All” and “Treat-None” strategies as baseline references (16).

### 2.8 Model interpretation

SHAP was used to interpret the prediction results of the models. SHAP is a model-agnostic method for explaining machine learning predictions. It is based on Shapley values, which quantify the contribution of each feature to a given prediction. In this way, SHAP helps to explain the decision-making process of the model, especially in interpreting complex “black-box” models (17).

We used the weighted ensemble model as the explainer and calculated SHAP values across the entire training dataset. The SHAP analysis included feature importance ranking (summary bar plot), feature impact distribution (summary dot plot), individual-level explanation (waterfall plot), feature dependence visualization (dependence plot), cross-center population importance comparison (forest plot); direction consistency analysis (Cleveland dot plot); subgroup difference analysis (dependence plots).

Feature importance was evaluated by the mean absolute SHAP value of each input variable, indicating its global influence on the model’s output. The summary dot plot displayed the distribution of SHAP values for each feature across all samples, along with feature values to reveal positive or negative directional impact.

An individual-level explanation was demonstrated using a waterfall plot for the second patient in the dataset, showing the cumulative contribution of each feature to the model’s prediction. Dependence plots were generated for key features to explore the functional relationships and interactions between feature values and SHAP values.

Cross-center population importance comparison (forest plot). For each feature, we computed the mean absolute SHAP value (mean |SHAP|) in the overall sample, the NHANES subcohort, and the hospital subcohort. We then calculated the between-center difference Δ and obtained a 95% bootstrap confidence interval for Δ. Results are displayed as a horizontal forest plot ordered by Δ; error bars that cross the zero line indicate no significant difference. Direction consistency analysis (Cleveland dot plot). For each feature, we calculated the proportion of instances with SHAP >0 in three groups (overall, NHANES, hospital). This proportion indicates whether higher feature values tend to increase or decrease the predicted risk. Plotting and connecting the three points allows a visual assessment of directional consistency across populations. Subgroup difference analysis (dependence plots). We stratified the data by sex (female vs. male) and age (<60 vs. ≥60 years), computed mean SHAP within each subgroup, and calculated the between-subgroup difference Δ. Features with larger differences are summarized using horizontal forest-style plots to highlight subgroup-specific explanatory strength.

### 2.9 Web-based calculator

An online risk prediction calculator was developed based on the FastAPI framework. The model consists of four base learners (Random Forest, LightGBM, XGBoost, and CatBoost) and a weighted ensemble model, each loading pretrained model files for inference. After users input clinical indicators via the web interface, the backend automatically calculates derived variables (NLR, SII, Age_group) and interaction terms (BMI × Age, Hemoglobin × Creatinine, Albumin × ALT). These features are then standardized and passed into the models to obtain prediction probabilities, which are finally aggregated by the ensemble model to output the overall risk probability.

The system has been deployed on a public server and is accessible online for real-time prediction: https://huggingface.co/spaces/FYZhouLab/Low_muscle_mass.

### 2.10 Statistical software

The logistic regression, KNN, random forest, and SVM models were implemented using the scikit-learn library. The XGBoost model was built with the xgboost library, LightGBM was implemented using the lightgbm library, and the CatBoost model was constructed with the catboost library.

## 3 Results

### 3.1 Baseline characteristics

**Figure 1:**
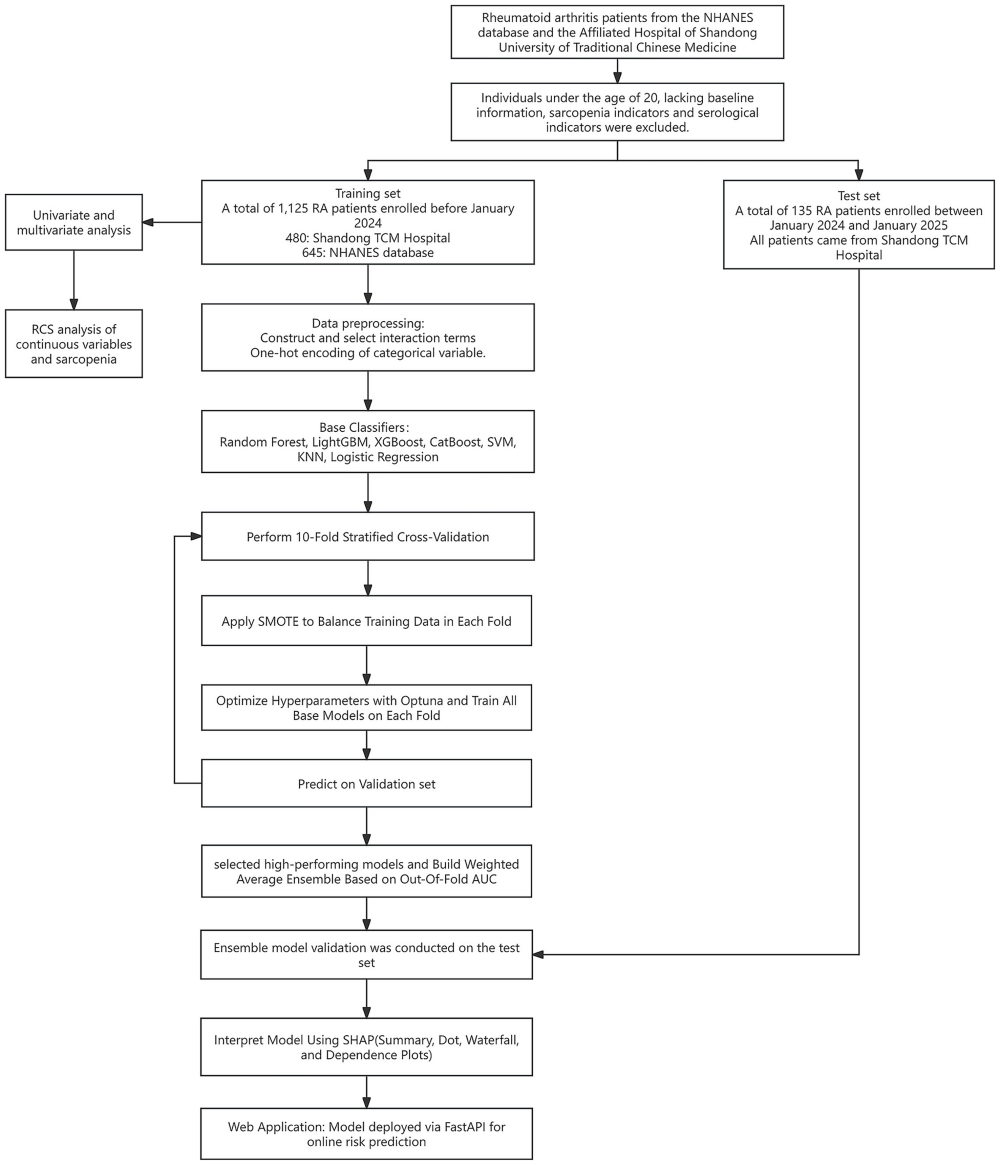
Flowchart of model development and verification.

A total of 1,260 individuals with RA were included in this study, of whom 615 were from the SHUTCM and 645 from the NHANES database. In the overall dataset, 74.1% of participants were female, and 25.9% were male. Baseline characteristics were stratified by low muscle mass status. Significant differences were observed between the low muscle mass group (G1) and the non-low muscle mass group (G2) across several baseline variables. The mean age of the G1 group was 55.44 ± 0.82 years, significantly higher than that of the G2 group (51.59 ± 0.38 years, *p* < 0.001). In addition, the hemoglobin level in G1 was slightly lower than that in G2 (*p* = 0.030). Detailed baseline characteristics are presented in Table 1, and the study design is illustrated in Figure 1.

**Table 1:** Baseline characteristics.

Characteristic	N1	Overall	G1	G2	*p*-value
Gender					0.77
Female	934	934 (74.1%)	172 (75.1%)	762 (73.9%)	
Male	326	326 (25.9%)	57 (24.9%)	269 (26.1%)	
Age group					<0.001
Age <60	931	931 (73.9%)	146 (63.8%)	785 (76.1%)	
Age >60	329	329 (26.1%)	83 (36.2%)	246 (23.9%)	
Age	1,260	52.29 ± 0.34	55.44 ± 0.82	51.59 ± 0.38	<0.001
Cholesterol	1,260	4.91 (4.29–5.69)	4.89 (4.27–5.69)	4.94 (4.29–5.69)	0.648
Lymphocyte	1,260	1.79 (1.40–2.30)	1.84 (1.41–2.40)	1.77 (1.38–2.30)	0.201
Neutrophil	1,260	3.80 (3.00–4.80)	4.10 (3.10–5.00)	3.78 (3.00–4.72)	0.075
Hemoglobin	1,260	13.20 (12.20–14.30)	12.90 (11.10–14.40)	13.30 (12.30–14.20)	0.003
Platelet	1,260	254.00 (212.00–308.25)	261.00 (211.00–320.00)	253.00 (213.00–305.50)	0.281
Albumin	1,260	41.00 (39.00–43.00)	39.50 (38.00–42.00)	41.40 (39.25–43.25)	<0.001
ALT	1,260	19.00 (14.00–26.00)	18.00 (13.00–25.00)	19.00 (14.00–26.00)	0.452
AST	1,260	21.00 (18.00–26.00)	21.00 (17.00–27.00)	21.00 (18.00–26.00)	0.459
UREA	1,260	4.64 (3.86–5.71)	4.64 (3.89–5.71)	4.65 (3.81–5.71)	0.737
Uric acid	1,260	283.00 (232.00–345.00)	261.70 (212.00–333.10)	287.00 (236.50–345.00)	0.004
Creatinine	1,260	61.00 (49.00–77.79)	54.00 (44.20–70.72)	61.88 (50.39–78.68)	<0.001
NLR	1,260	2.10 (1.53–2.86)	2.11 (1.55–2.94)	2.09 (1.53–2.84)	0.834
SII	1,260	532.93 (367.68–776.12)	575.92 (363.84–827.44)	523.62 (370.15–772.57)	0.389
BMI	1,260	26.72 (23.47–31.13)	25.29 (20.61–32.96)	26.98 (24.01–30.90)	<0.001

ALT, alanine aminotransferase; AST, aspartate aminotransferase; UREA, serum urea nitrogen; NLR, neutrophil to lymphocyte ratio; SII, Systemic Immune Inflammation Index; BMI, body mass index. G1: low muscle mass group; G2: non-low muscle mass group.

To compare the baseline characteristics of training set between the two study centers, the K–S test was performed for all non-outcome variables. The results indicated that several variables exhibited significant distributional differences between the two centers. Detailed information is provided in Supplementary Table 1.

### 3.2 Independent risk factors and nonlinear relationships

To investigate the association between clinical variables and low muscle mass, we performed univariate logistic regression (Table 2) and multivariate logistic regression analyses (Table 3) using the training dataset. In the univariate analysis, age, neutrophil count, albumin, creatinine, SII, and older age group were significantly associated with low muscle mass. In the multivariate analysis, age, albumin, creatinine, and neutrophil count remained independently associated with low muscle mass.

**Table 2:** Univariate logistic regression.

Variable	OR	95% CI	*p*-value
Albumin	0.889	(0.85, 0.93)	<0.001
Age	1.030	(1.02, 1.04)	<0.001
Age_group	1.857	(1.34, 2.58)	<0.001
Neutrophil	1.118	(1.02, 1.22)	0.014
Creatinine	0.992	(0.99, 1.00)	0.030
SII	1.000	(1.00, 1.00)	0.040
NLR	1.106	(0.98, 1.25)	0.103
Uric_acid	0.999	(1.00, 1.00)	0.213
Platelet	1.001	(1.00, 1.00)	0.218
Lymphocyte	1.094	(0.89, 1.35)	0.406
Hemoglobin	0.982	(0.92, 1.04)	0.563
BMI	0.994	(0.97, 1.02)	0.611
AST	0.997	(0.98, 1.01)	0.614
Cholesterol	0.982	(0.85, 1.13)	0.806
ALT	0.999	(0.99, 1.01)	0.874
UREA	1.005	(0.93, 1.09)	0.899
Gender	1.010	(0.72, 1.42)	0.952

ALT, alanine aminotransferase; AST, aspartate aminotransferase; UREA, serum urea nitrogen; NLR, neutrophil to lymphocyte ratio; SII, Systemic Immune Inflammation Index; BMI, body mass index; 95% CI, 95% confidence interval.

**Table 3:** Multivariate logistic regression.

Variable	OR	95% CI	*p*-value
Intercept	2.284	(0.28, 18.58)	0.440
Albumin	0.903	(0.86, 0.94)	<0.001
Age	1.036	(1.01, 1.06)	<0.001
Creatinine	0.989	(0.98, 1.00)	0.003
Neutrophil	1.182	(1.04, 1.34)	0.008
SII	0.999	(1.00, 1.00)	0.365
Age_group >60	0.999	(0.60, 1.67)	0.999

SII, Systemic Immune Inflammation Index; 95% CI, 95% confidence interval.

**Figure 2:**
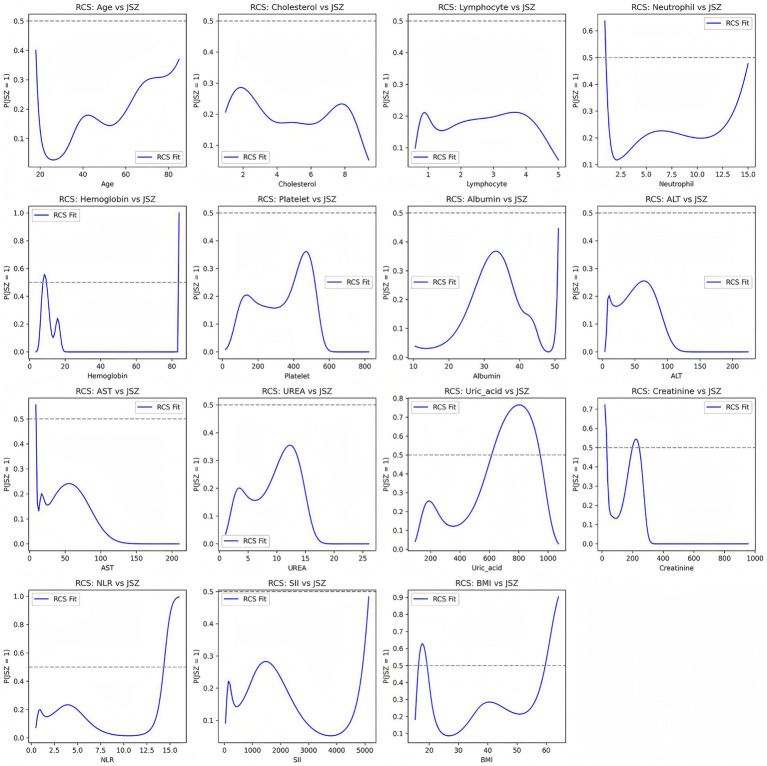
Nonlinear associations between continuous variables and risk of low muscle mass based on restricted cubic spline (RCS) models.

To further explore potential nonlinear relationships between continuous variables and risk of low muscle mass, we used RCS models. The results, presented in Figure 2, showed that some variables exhibited marked nonlinear associations with low muscle mass, suggesting that traditional linear models may underestimate the true impact of these factors.

Besides, in the SHUTCM clinical dataset, we quantified the associations between hematology-derived inflammatory indices (NLR, SII) and acute-phase reactants (ESR, CRP) using Spearman’s rank correlation. All four correlations were positive and statistically significant, but the effect sizes were in the weak range, indicating that SII and NLR partially reflect systemic inflammatory burden yet cannot fully substitute for the acute-phase response represented by CRP/ESR (Supplementary Table 2).

### 3.3 Model development and performance comparison

Given the limited number of features included in this study (17 in total), no explicit variable selection was performed to avoid potential information loss. All features were retained for model development. During model training, ensemble learning and cross-validation were applied to reduce the risk of overfitting, and SHAP analysis was used to assess feature importance.

We developed and compared eight classification algorithms, including Random Forest, LightGBM, XGBoost, CatBoost, SVM, KNN, Logistic Regression, and an AUC-weighted ensemble model. Among the base learners, CatBoost (AUC = 0.772), LightGBM (AUC = 0.768), Random Forest (AUC = 0.766), and XGBoost (AUC = 0.753) showed better performance. Cross-validation results confirmed the stability of these findings. Given the superior predictive performance of tree-based models, we constructed a weighted ensemble model based on the AUC scores of four tree-based learners. The weighted ensemble model achieved the best overall performance, with an AUC of 0.921, significantly outperforming all individual models. The average performance metrics of each machine learning model across 10-fold cross-validation are presented in Table 4, while the Out-of-Fold (OOF) performance metrics are summarized in Table 5.

**Table 4:** Average performance metrics of each machine learning model based on 10-fold cross-validation.

Model	Accuracy	AUC	F1	Precision	Recall	BrierScore
RandomForest	0.820 ± 0.044	0.776 ± 0.047	0.546 ± 0.091	0.506 ± 0.109	0.603 ± 0.097	0.174 ± 0.023
LightGBM	0.803 ± 0.070	0.765 ± 0.071	0.526 ± 0.089	0.498 ± 0.136	0.608 ± 0.156	0.153 ± 0.031
XGBoost	0.804 ± 0.068	0.766 ± 0.065	0.532 ± 0.084	0.505 ± 0.157	0.603 ± 0.082	0.312 ± 0.082
CatBoost	0.823 ± 0.041	0.773 ± 0.043	0.539 ± 0.071	0.524 ± 0.098	0.587 ± 0.126	0.146 ± 0.029
SVM	0.732 ± 0.117	0.711 ± 0.070	0.476 ± 0.073	0.414 ± 0.140	0.643 ± 0.146	0.173 ± 0.029
KNN	0.662 ± 0.132	0.637 ± 0.050	0.394 ± 0.043	0.333 ± 0.116	0.608 ± 0.206	0.267 ± 0.027
LogisticRegression	0.657 ± 0.113	0.651 ± 0.035	0.416 ± 0.031	0.324 ± 0.083	0.674 ± 0.165	0.232 ± 0.010

Accuracy, overall proportion of correctly classified cases; AUC, area under the curve; F1, harmonic mean of precision and recall; Precision, proportion of true positives among predicted positives; Recall (sensitivity), proportion of true positives among actual positives.

**Table 5:** Out-of-fold performance metrics of machine learning models based on 10-fold cross-validation.

Model	Accuracy	AUC	F1	Precision	Recall	BrierScore
RandomForest	0.809	0.766	0.508	0.466	0.558	0.174
LightGBM	0.773	0.768	0.489	0.407	0.613	0.153
XGBoost	0.782	0.753	0.464	0.411	0.533	0.312
CatBoost	0.835	0.772	0.492	0.539	0.452	0.146
SVM	0.685	0.710	0.41	0.307	0.618	0.173
KNN	0.528	0.637	0.347	0.230	0.709	0.267
LogisticRegression	0.64	0.652	0.370	0.268	0.598	0.232
Ensemble model	0.859	0.921	0.651	0.578	0.744	0.094

Accuracy, overall proportion of correctly classified cases; AUC, area under the curve; F1, harmonic mean of precision and recall; Precision, proportion of true positives among predicted positives; Recall (sensitivity), proportion of true positives among actual positives.

**Figure 3:**
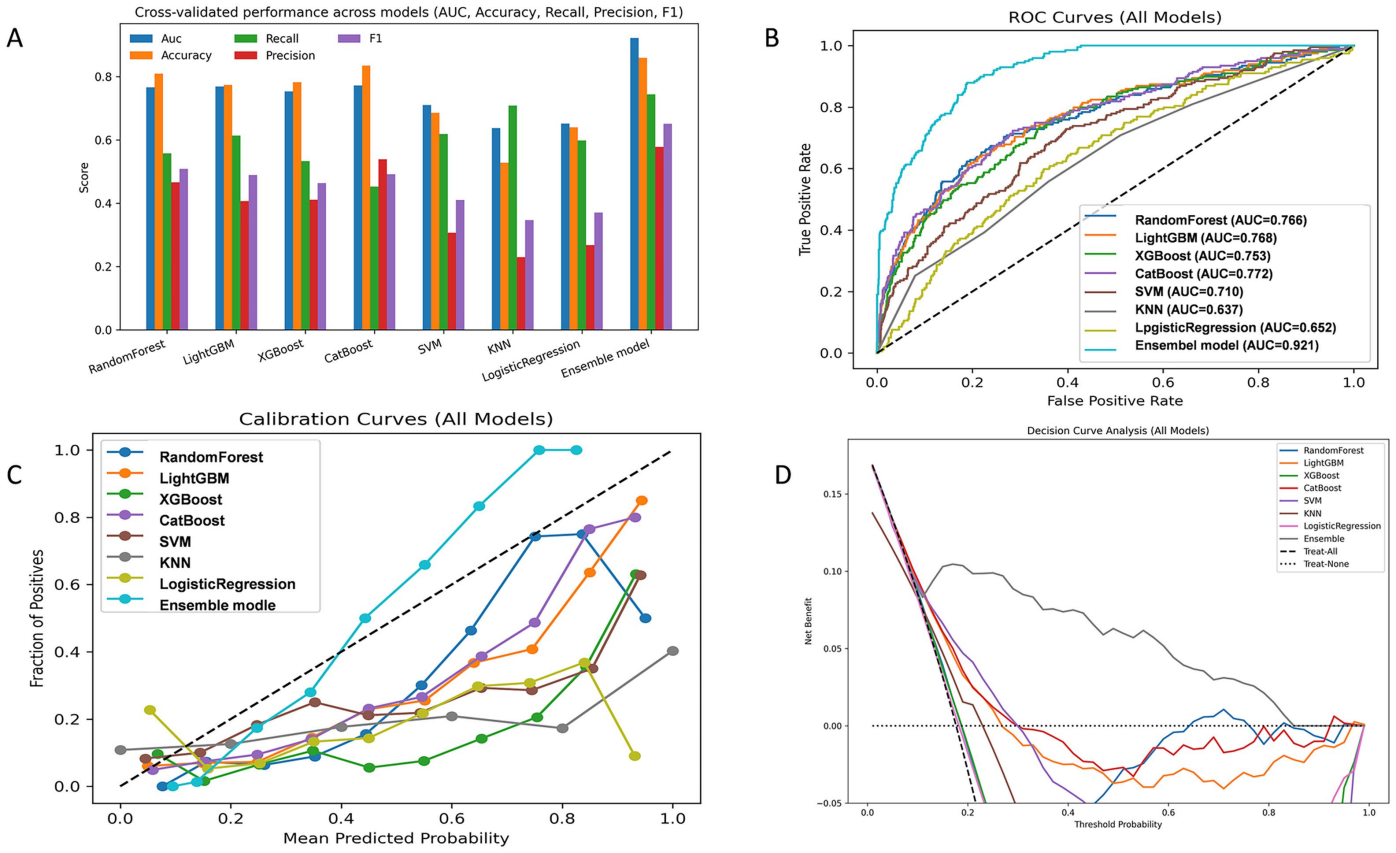
Model performance evaluation in the validation sets. **(A)** Grouped bar chart comparing key evaluation metrics (AUC, Accuracy, Recall, Precision, F1) across candidate models. **(B)** Receiver operating characteristic (ROC) curve. **(C)** Calibration curve assessing agreement between predicted and observed probabilities. **(D)** Decision curve analysis (DCA) evaluating clinical net benefit.

Calibration curves showed that the ensemble model’s predicted probabilities closely matched actual event rates, indicating better calibration than other models. Decision curve analysis showed that the ensemble model achieved higher net clinical benefit across a wide range of threshold probabilities, suggesting greater clinical utility. Figure 3 presents the performance of the models on the validation set.

**Figure 4:**
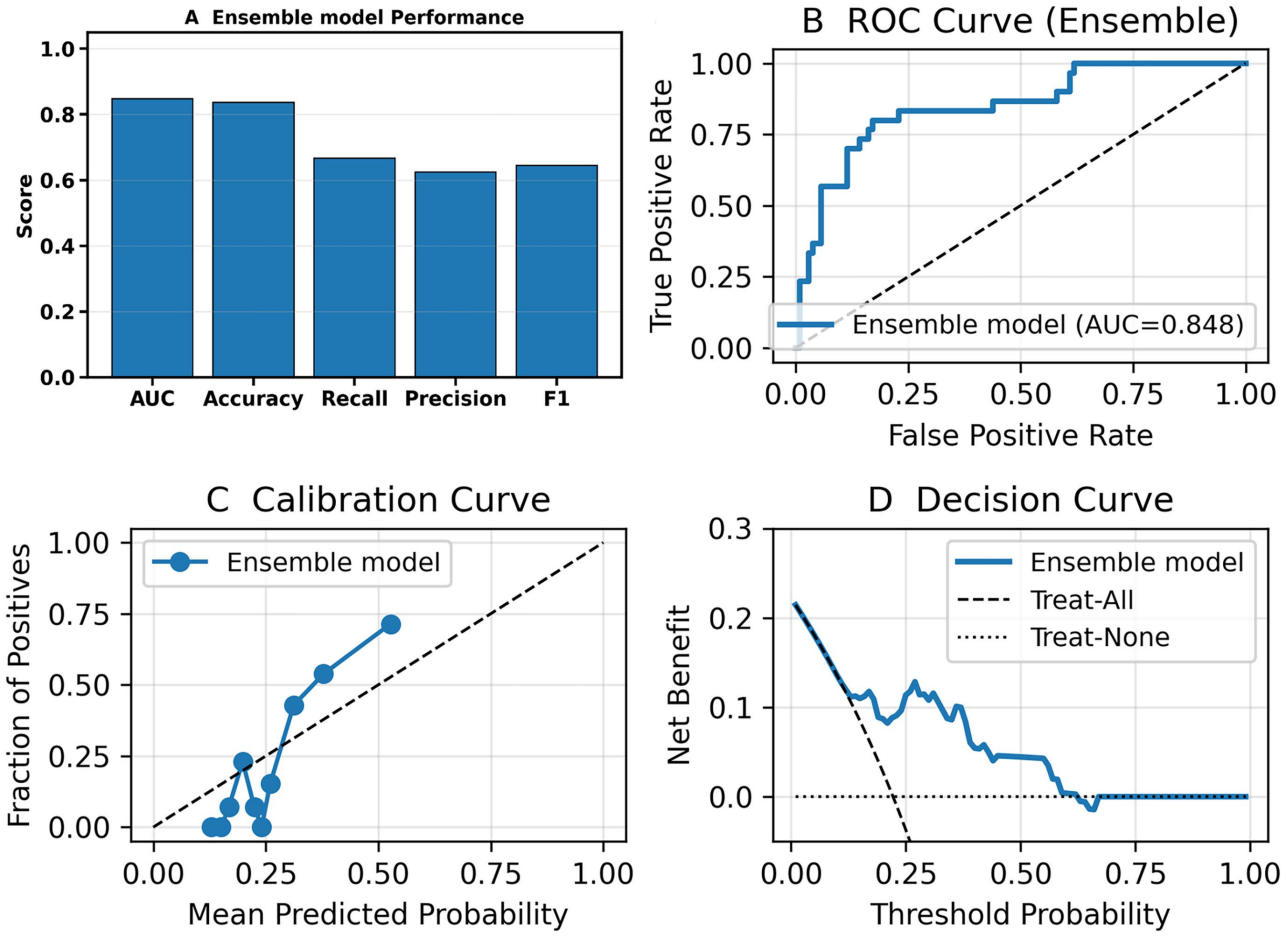
Performance of the ensemble model on the independent test set. **(A)** Bar chart summarizing overall performance of the ensemble model—AUC, Accuracy, Recall, Precision, and F1. **(B)** Receiver operating characteristic (ROC) curve showing the discriminative ability of the model. **(C)** Calibration curve demonstrating agreement between predicted probabilities and observed outcomes. **(D)** Decision curve analysis (DCA) showing the net clinical benefit of the ensemble model compared with the “treat-all” and “treat-none” strategies.

To evaluate the generalizability of the ensemble model, we conducted validation on the test set. Figure 4 presents the performance of the models on the validation set. On the independent test set, the ensemble model demonstrated robust discriminatory ability, with an AUC of 0.848 (Figure 4B). As shown in Figure 4A, the model achieved consistent performance across multiple evaluation metrics, including Accuracy, Recall, Precision, and F1 score. The calibration curve (Figure 4C) indicated that the predicted probabilities were reasonably aligned with the observed outcomes, although some degree of underestimation was observed at higher probability ranges. Decision curve analysis (Figure 4D) further showed that the ensemble model provided a greater net clinical benefit compared with the “treat-all” and “treat-none” strategies, particularly within the threshold probability range of 0.1–0.4, suggesting good clinical applicability of the model. Performance of the ensemble model on the test set are shown in Table 6.

**Table 6:** Performance of the ensemble model on the test set.

Model	Accuracy	AUC (95% CI)	F1	Precision	Recall	BrierScored	BestThresholdUsed	Permutation *p*-value
Ensemble model	0.837	0.848 (0.770, 0.923)	0.645	0.625	0.667	0.133	0.326	<0.001

Accuracy, overall proportion of correctly classified cases; AUC, area under the curve; F1, harmonic mean of precision and recall; Precision, proportion of true positives among predicted positives; Recall (sensitivity), proportion of true positives among actual positives.

### 3.4 Model interpretation

**Figure 5:**
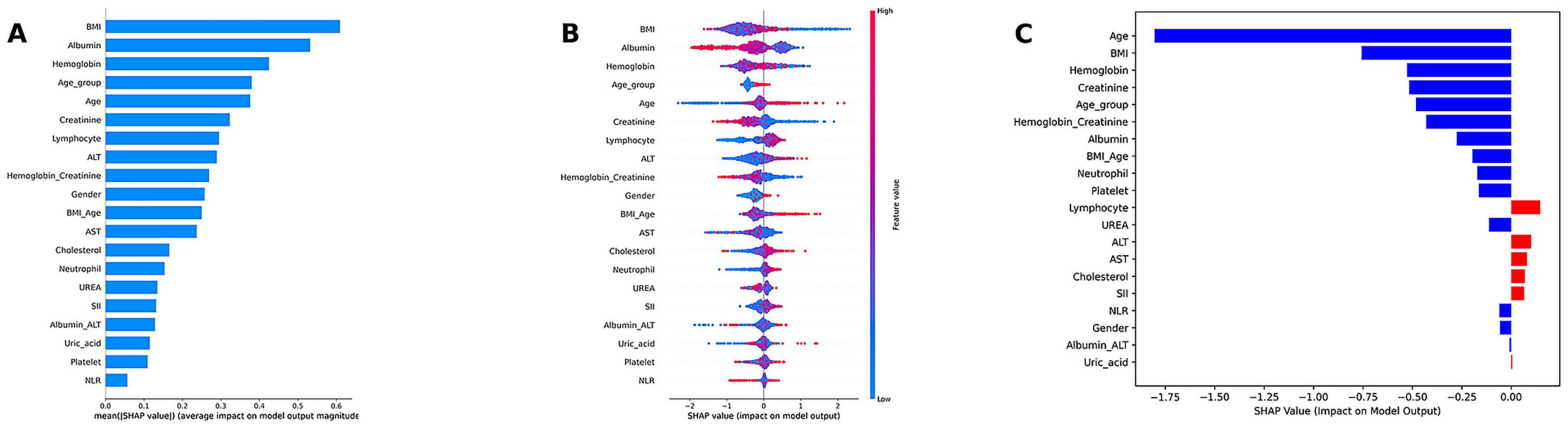
Model interpretation of the weighted ensemble model using SHAP. **(A)** SHAP summary bar plot illustrating the global importance of each feature. **(B)** SHAP summary dot plot, showing the global importance, direction, and distribution of features. **(C)** SHAP waterfall plot for an individual case.

Due to the complex ensemble structure and nonlinear interactions of the weighted ensemble model, it is inherently less interpretable and considered a “black-box” model. To address this limitation, we applied the SHAP method, which quantifies the contribution of each feature to the model’s prediction, enabling interpretation of the model output. SHAP analysis results are visualized in Figure 5.

Model interpretation was conducted at both the global (feature-level) and local (individual-level) levels. At the global level, we used SHAP summary bar plots (Figure 5A) and dot plots (Figure 5B) to evaluate the overall contribution of each feature to the model. The bar plot ranks features based on their mean absolute SHAP values, revealing that BMI, albumin, hemoglobin, age, and creatinine were the top five contributors to the model’s predictions. The SHAP summary dot plot provides a visual representation of the direction and magnitude of each feature’s impact on the prediction. It showed that higher levels of BMI, creatinine, albumin, and hemoglobin were associated with a lower predicted risk of low muscle mass, while older age, lymphocyte count, and neutrophil count were associated with increased risk. At the local level, we used SHAP waterfall plots (Figure 5C) to visualize the model’s decision process for individual patients. A waterfall plot was generated for the second patient in the test set, showing the contribution of each feature (sorted in descending order of absolute SHAP value) to the final prediction.

**Figure 6:**
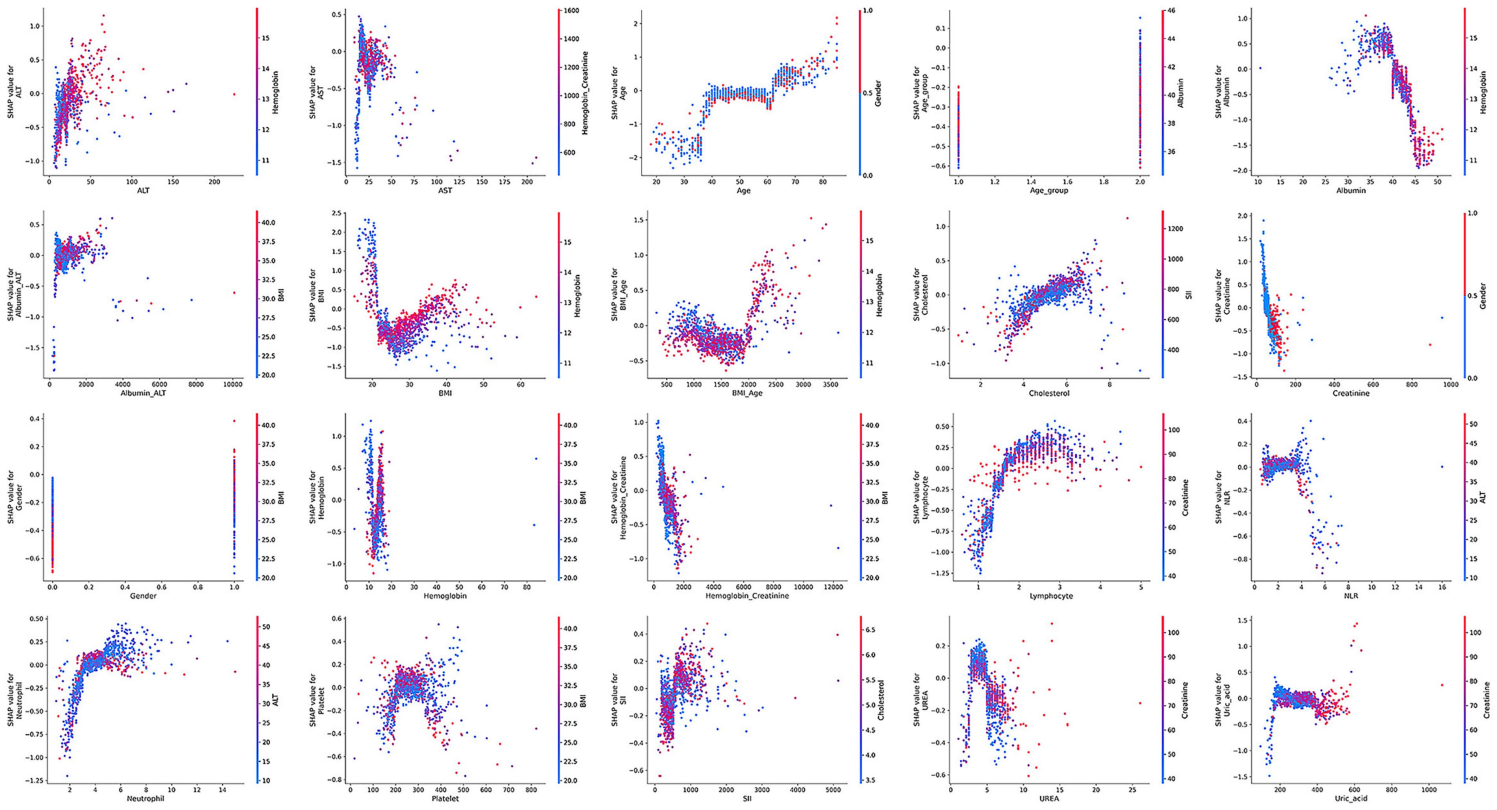
SHAP dependence plots for clinical features in the ensemble model.

SHAP dependence plots (Figure 6) further illustrated how individual features influenced model predictions. Features with SHAP values greater than zero were positively associated with the model’s prediction of low muscle mass.

**Figure 7:**
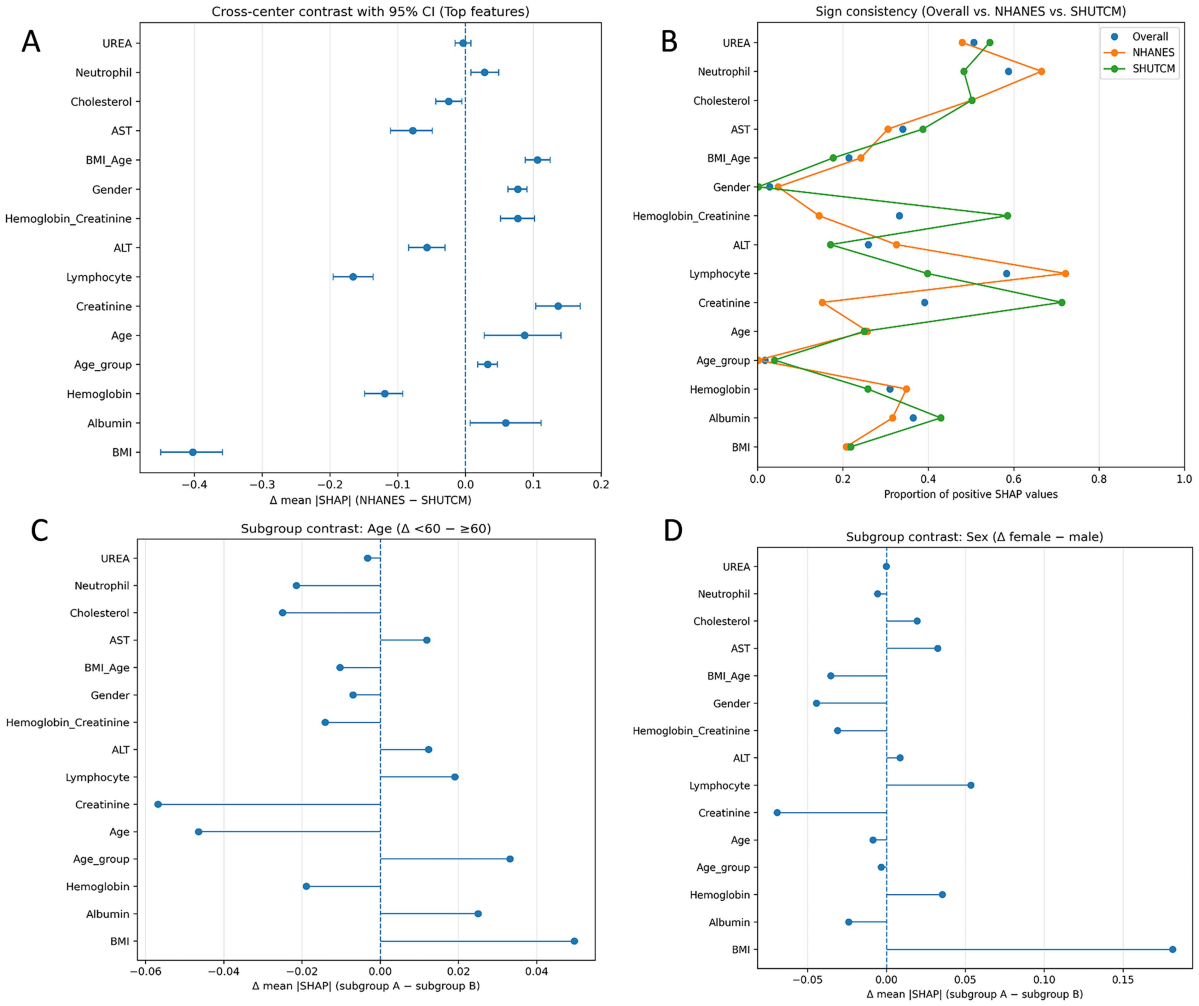
Cross-population explainability analyses. **(A)** Cross-center population importance comparison (forest plot). Top features are ranked by overall mean |SHAP|. Points show Δ (mean|SHAP|) = NHANES − SHUTCM; horizontal error bars denote 95% bootstrap CIs; the vertical line marks no difference (Δ = 0). Negative Δ indicates greater explanatory strength in the hospital cohort. **(B)** Direction consistency analysis (Cleveland dot plot). For each feature, points indicate the proportion of instances with SHAP >0 in the overall sample, NHANES, and SHUTCM. **(C)** Subgroup difference analysis by age. Forest-style summary of Δ (mean|SHAP|) = Age <60 – Age ≥60. **(D)** Subgroup difference analysis by sex. Δ (mean|SHAP|) = female − male.

Cross-center population importance comparison (Figure 7A). Among the top-ranked features by overall mean SHAP, we observed clear between-cohort differences. BMI had a markedly negative Δ (mean|SHAP|, NHANES − SHUTCM) with a 95% CI not crossing zero, indicating greater explanatory strength in the hospital cohort. In contrast, albumin, creatinine, and age/age_group showed positive Δ with CIs not crossing zero, implying larger contributions in NHANES. Lymphocyte count and ALT also tended to favor the hospital side (negative Δ), whereas several metabolic/inflammatory variables exhibited small, zero-crossing intervals, suggesting more portable signals across populations. This pattern is consistent with differences in case mix and laboratory measurement ranges between a population survey and a tertiary care setting. Direction consistency analysis (Figure 7B). Most core features displayed broadly consistent directions across populations (proportions with SHAP >0 well away from 0.5). Albumin predominantly showed a negative association, while age and creatinine were largely positive. BMI showed mixed and non-linear behavior; in the hospital cohort its contribution strengthened at higher values, consistent with the threshold or steep-rise patterns in the dependence plots. Overall, the directionality supports a nutrition–inflammation–muscle-mass axis as a cross-population stable signal, while the population-amplified effect of BMI warrants attention and potential local calibration at deployment. Subgroup difference analysis (Figures 7C,D). Using Δ (<60 − ≥60) for the age contrast, creatinine and age itself contributed more strongly among older participants (negative Δ), whereas BMI and albumin were relatively more influential in the <60 group (positive Δ). This suggests effect modification by age, with muscle-mass/renal-clearance markers more tightly linked to outcomes in older adults and weight/nutritional status contributing more among younger adults. For the sex contrast Δ (female − male), BMI showed greater explanatory strength in women (positive Δ), while creatinine was more important in men (negative Δ); other features differed only modestly. These patterns align with sex-specific body fat/muscle distribution and physiological thresholds, supporting subgroup-aware thresholds and tailored risk communication.

### 3.5 Implementation of the web calculator

We successfully developed and deployed an online sarcopenia risk prediction calculator based on the FastAPI framework. The system integrates four base learners—Random Forest, LightGBM, XGBoost, CatBoost, and weighted ensemble model to generate individualized risk predictions. Upon entering relevant clinical variables via the web interface, the system automatically computes derived features (e.g., NLR, SII, Age_group) and interaction terms (e.g., BMI × Age, Hemoglobin × Creatinine, Albumin × ALT). All inputs are then standardized and passed into each model for inference. The final risk probability score is output by the ensemble model. The system is currently deployed on the Hugging Face Spaces platform and supports real-time online access and prediction: https://huggingface.co/spaces/FYZhouLab/Low_muscle_mass.

## 4 Discussion

This study developed a clinical prediction model that leverages routinely collected diagnostic and treatment data to provide preliminary screening for muscle mass in RA patients. The model may reduce the need for routine sarcopenia screening procedures and enable more targeted diagnostic evaluation for individuals at high risk of sarcopenia.

In the final model we developed, BMI, albumin, hemoglobin, age, and creatinine were identified as the five most important features. BMI and serum albumin are commonly considered surrogate markers of nutritional status and muscle mass. However, patients with RA often exhibit elevated systemic inflammatory burden and additional physiological impairments, which may confer additional clinical significance to these indicators within the RA population.

In the final model, SHAP dependence analysis indicated that while low BMI exhibited a stronger association with low muscle mass, excessively high BMI values were also positively associated with increased risk. Low BMI may be indicative of rheumatoid cachexia, while high BMI may reflect sarcopenic obesity in patients with RA. Sarcopenic obesity refers to a pathological body composition characterized by the coexistence of reduced muscle mass and excessive fat accumulation, and it has a relatively high prevalence among individuals with RA (18). Unlike traditional simple obesity, RA-related sarcopenic obesity typically involves a dual alteration: a decrease in lean body mass and an increase in fat mass. The condition of reduced lean mass in RA is also referred to as rheumatoid cachexia (19), which is often driven by chronic inflammation that impairs both the synthesis and degradation of skeletal muscle proteins (20). This state is associated with increased disease activity and higher mortality risk (21–23). Therefore, although some RA patients may present with elevated BMI, chronic inflammation-induced loss of lean mass may lead to the coexistence of low muscle mass and high BMI, highlighting the need for careful interpretation of BMI in this population.

Hemoglobin is a sensitive indicator of both inflammation-related anemia and nutritional status (24–26). In patients with RA, hemoglobin levels are closely associated not only with disease activity but also with tissue damage caused by chronic inflammation (27). Multiple studies have demonstrated a significant correlation between low hemoglobin levels and clinical joint damage, independent of traditional disease activity markers. Hemoglobin has been proposed as an independent risk factor for predicting joint and other tissue damage (28, 29). Chronic anemia associated with RA is considered one of the common comorbidities of the disease (30), and its underlying mechanisms may involve reduced red blood cell lifespan, pathological iron homeostasis driven by hepcidin, and a diminished response to erythropoietin (31). Research suggests that chronic anemia may contribute to the development and progression of low muscle mass by impairing oxygen delivery to muscle tissue (32), providing a potential pathophysiological explanation for the decline in muscle mass observed in RA patients.

Serum albumin is the most abundant protein in plasma and serves as a key indicator of nutritional status. Recent studies have shown that malnutrition can lead to decreased serum albumin levels, accelerate the loss of lean body mass, and subsequently contribute to the development of low muscle mass—a mechanism that may be closely associated with functional decline and reduced muscle strength or mass in older adults (33). In addition to reflecting nutritional reserve, serum albumin is a negative acute-phase reactant (34): its concentration decreases when IL-6 driven hepatic acute-phase signaling is activated. Clinically, albumin fluctuations are closely tied to outcomes in critical illness. In rheumatoid arthritis (RA), cytokine-mediated inflammation, predominantly IL-6, IL-1 and TNF-*α*, engages the gp130–STAT3 pathway, shifting hepatocyte protein synthesis toward positive acute-phase proteins and down-regulating albumin (35–37); capillary leak, hemodilution, and catabolic effects further lower circulating levels. This mechanistic framework explains the inverse relation between albumin and inflammatory activity and the frequent hypoalbuminemia in active RA observed clinically (38, 39).

Lower serum creatinine reflects reduced muscle mass, whereas higher values can also reflect impaired renal clearance, this dual dependence explains why creatinine alone is an imperfect proxy for sarcopenia. In rheumatoid arthritis (RA), chronic cytokine-driven inflammation (TNF-α, IL-6) promotes rheumatoid cachexia, accelerating muscle protein breakdown, reducing synthesis, and predisposing to low muscle mass, thereby linking inflammatory activity to creatinine declines via loss of muscle substrate (40, 41). To disentangle muscle from kidney effects, several studies propose the sarcopenia index (SI = serum creatinine/serum cystatin C × 100) or the creatinine-to-cystatin C ratio, leveraging the fact that cystatin C is largely independent of muscle mass. These indices show promising diagnostic and prognostic performance for low muscle mass across cohorts (42–45). Our findings are consistent with this biology: in an RA population where inflammation-driven muscle wasting is prevalent, creatinine provide clinically useful signals for identifying individuals at risk of low muscle mass, while also acknowledging renal function as a key confounder (46).

We acknowledge several limitations in this study. First, this analysis used multicenter data. Because the single-center cohort from the Affiliated Hospital of Shandong University of Traditional Chinese Medicine did not provide enough events for machine-learning training, and because RA severity and case-mix differ between community participants and hospital patients, we augmented the dataset with NHANES, a nationally representative U.S. Health Examination Survey, and adopted a dual-source design (“population survey and hospital”) to improve transportability across community and clinical settings. Results from the K–S test indicated that most non-outcome variables exhibited significant distributional differences between the two centers. The cross-center heterogeneity we observe constitutes a domain shift that can influence both bias and transportability. First, spectrum effects arise when case severity and prevalence differ by site: discrimination (e.g., AUC) may remain acceptable while calibration drifts, causing misestimation of absolute risk and threshold-dependent bias (PPV/NPV, net benefit) when a model trained in one spectrum is applied to another. Second, measurement shifts such as different laboratory ranges, assay platforms, or coding practices, can change the apparent effect size of predictors, creating center-dependent signals that degrade portability if not recognized. To assess potential bias and generalizability, we reported performance on a time-split external test set, showing that the ensemble retained probability calibration and net clinical benefit despite distributional shifts. We also added cross-population SHAP analyses (Figures 7A–D) to quantify differences in feature contributions between NHANES and the hospital cohort and to identify signals that are portable versus center-dependent. To minimize bias and preserve generalizability, we recommend: (1) site-specific recalibration (isotonic/Platt) and cut-point tuning using a small local sample before deployment; (2) prospective monitoring of calibration and decision metrics with drift checks (e.g., PSI/K–S) and scheduled re-assessment; and (3) if future settings diverge more substantially, consider re-weighting, domain-adaptation, or hierarchical/multi-source training as extensions.

Second, the NHANES database lacks RA-specific disease activity measures such as DAS28, RAPID-3, and, in certain cycles, C-reactive protein (CRP) and erythrocyte sedimentation rate (ESR). These indicators are frequently used in clinical practice and are closely associated with long-term outcomes in RA patients, including cardiovascular mortality. This limitation arises from the design of the NHANES database, which is intended for population-level health surveillance rather than disease-specific clinical research. As a result, key components required to calculate conventional RA disease activity scores—such as tender and swollen joint counts, patient-reported outcomes, CRP, and ESR—were unavailable, preventing us from incorporating direct measures of RA disease activity into the predictive model. To compensate for this limitation, we included the NLR and SII as indirect indicators of RA disease activity. Previous studies have demonstrated that NLR is positively correlated with ESR and CRP in RA populations (47), and that SII is associated with DAS28-ESR and DAS28-CRP (48, 49). Several additional studies also support the strong association of NLR and SII with RA disease activity (47, 50). Although NLR and SII can partially reflect systemic inflammation and disease activity in RA, they remain surrogate markers and have inherent limitations. Consistent with prior literature, both NLR and SII showed positive, statistically significant correlations with CRP and ESR in our dataset, indicating that these indices partially track systemic inflammatory burden. However, the effect sizes were in the weak range, aligning with published evidence that NLR/SII correlate with disease activity but do not fully substitute for canonical markers or composite scores. We acknowledge the predictive value of traditional inflammatory markers such as CRP and ESR, as well as disease activity scores like DAS28, in the context of RA-associated low muscle mass. Therefore, future work should incorporate RA-specific disease activity indicators to further optimize and validate the predictive model.

Moreover, RA disease activity typically fluctuates over time and in response to treatment, rather than remaining constant (51). NLR and SII are highly sensitive to changes in RA disease activity; therefore, their elevation during periods of active disease may lead to a higher likelihood of RA patients being classified as high-risk for low muscle mass by the model. This study was based on cross-sectional data, capturing only the baseline values of NLR and SII at a single time point. As a result, the model reflects inflammation levels at a specific moment, without accounting for the longitudinal variation in RA disease activity. In future research, we aim to incorporate RA-specific disease activity indicators and their temporal dynamics into the predictive model to better represent disease progression over time.

We developed an interpretable machine learning model to predict the risk of low muscle mass in patients with RA. The final weighted ensemble model demonstrated excellent predictive performance. Future research should perform prospective external validation in an independent center to further evaluate model transportability and calibration.

## Data Availability

The data analyzed in this study is subject to the following licenses/restrictions: The NHANES data analyzed in this study are publicly available from the National Health and Nutrition Examination Survey (NHANES) website. Clinical data from the Affiliated Hospital of Shandong University of Traditional Chinese Medicine are not publicly available due to patient privacy and institutional restrictions. De-identified data underlying this study may be made available to qualified researchers from the author upon reasonable request. Access will require IRB/ethics approval at the requester’s institution, a signed data-use agreement (DUA) with the participating sites, and assurances that no attempts will be made to re-identify participants or to transfer data outside approved jurisdictions. Requests to access these datasets should be directed to FZ, zhoufeiyue0898@163.com.

## Glossary

RA: Rheumatoid arthritis
AUC: Area under the receiver operating characteristic curve
SHAP: SHapley Additive exPlanation
DXA: Dual-energy X-ray absorptiometry
BIA: Bioelectrical impedance analysis
NHANES: National Health and Nutrition Examination Survey
SHUTCM: Affiliated Hospital of Shandong University of Traditional Chinese Medicine
NCHS: National Center for Health Statistics
SVM: Support vector machine
KNN: K-nearest neighbors
BMI: Body mass index
ALT: Alanine aminotransferase
AST: Aspartate aminotransferase
SII: Systemic Immune-Inflammation Index
NLR: Neutrophil-to-lymphocyte ratio
SMOTE: Synthetic Minority Over-sampling Technique
SMI: Skeletal muscle mass index
ASM: Appendicular skeletal muscle mass
K–S: Kolmogorov–Smirnov
ROC: Receiver operating characteristic
DCA: Decision curve analysis
RCS: Restricted cubic spline
