# Supplementary material for: Development and validation of an interpretable machine learning model for predicting low muscle mass in patients with rheumatoid arthritis: a multicenter study
Source: Front Med (Lausanne). 2025 Nov 19;12:1694320. doi: 10.3389/fmed.2025.1694320 (PMC12672488; doi:10.3389/fmed.2025.1694320)
Supplement: Supplementary file 1 [file Table_1.docx]

**Supplementary Table 1** K-S Analysis

| **Variable** | **KS_p_value** |
| --- | --- |
| Gender | <0.001 |
| Cholesterol | <0.001 |
| Lymphocyte | <0.001 |
| Neutrophil | <0.001 |
| Hemoglobin | <0.001 |
| Albumin | <0.001 |
| ALT | <0.001 |
| AST | <0.001 |
| UREA | <0.001 |
| Uric_acid | <0.001 |
| Creatinine | <0.001 |
| NLR | <0.001 |
| SII | <0.001 |
| BMI | <0.001 |
| Age | 0.032 |
| Age_group | 0.0977 |
| Platelet | 0.696 |

**Notes:**

**Abbreviations:** ALT, alanine aminotransferase; AST, aspartate aminotransferase; UREA, serum urea nitrogen; NLR, neutrophil to lymphocyte ratio; SII, Systemic Immune Inflammation Index; BMI: Body mass index.

**Supplementary Table 2** Spearman correlations between hematology-derived inflammatory indices (NLR, SII) and acute-phase reactants (CRP, ESR) in the external validation dataset

|  |  | Spearman_rho | p_value | 95%CI | N |
| --- | --- | --- | --- | --- | --- |
| NLR | CRP | 0.162 | <0.001 | (0.076,0.252) | 480 |
| NLR | ESR | 0.163 | <0.001 | (0.065,0.258) | 480 |
| SII | CRP | 0.238 | <0.001 | (0.150,0.326) | 480 |
| SII | ESR | 0.247 | <0.001 | (0.151,0.336) | 480 |

NLR: neutrophil to lymphocyte ratio; SII, Systemic Immune Inflammation Index; CRP, C-reactive protein; ESR, erythrocyte sedimentation rate; CI, confidence interval; N: Total count of the dataset
